# Risk factors for subclinical inflammation in children with familial mediterranean fever

**DOI:** 10.1186/1546-0096-12-S1-P77

**Published:** 2014-09-17

**Authors:** Alper Soylu, Meral Torun Bayram, Tufan Çankaya, Elçin Bora, Salih Kavukçu, Ayfer Ülgenalp, Mehmet Türkmen

**Affiliations:** 1 Pediatrics, Dokuz Eylül University Medical Faculty , İzmir, Turkey

## Introduction

Familial Mediterranean fever (FMF) is the most common autosomal-recessive inherited inflammatory disease characterized by attacks of painful inflammation. Some patients with FMF have subclinical inflammation persisting between the attacks. Persistent inflammation may lead to amyloidosis and other complications of chronic inflammation.

## Objectives

We aimed to identify the demographic, clinical and genetic risk factors for subclinical inflammation in children with FMF.

## Methods

The medical records of the children with FMF were evaluated retrospectively for acute phase response (APR) along with gender, age at the onset of symptoms and at the time of diagnosis, clinical signs and symptoms (fever, abdominal pain, chest pain, arthritis/arthralgia, myalgia, erysipelas like erythema, protracted febrile myalgia, response to colchicine), presence of amyloidosis and *MEFV* genotype. Patients with persistently elevated AFR between the attacks were considered to have subclinical inflammation. Patients with or without subclinical inflammation (Group 1 and Group 2, respectively) were compared for the parameters defined above. Independent risk factors for subclinical inflammation were identified by multivariate logistic regression analysis.

## Results

There were 105 children (male/female: 52/53) who were compliant on colchicine treatment. Subclinical inflammation was detected in 22 (20%) patients. Group 1 had significantly higher rate of myalgia, arthritis/arthralgia, erysipelas like erythema, amyloidosis, protracted febrile myalgia and M694V mutation compared to Group 2. However, only the presence of myalgia and erysipelas like erythema were found to be independent risk factors for subclinical inflammation (OR 9.8 and 5.9, respectively).

## Conclusion

Children with FMF who have myalgia and erysipelas like erythema during the attacks are particularly at risk for ongoing inflammation and should be closely monitored for subclinical inflammation even during attack free periods.

## Disclosure of interest

None declared.

